# Clostridial Spores for Cancer Therapy: Targeting Solid Tumour Microenvironment

**DOI:** 10.1155/2012/862764

**Published:** 2012-06-07

**Authors:** Brittany Umer, David Good, Jozef Anné, Wei Duan, Ming Q. Wei

**Affiliations:** ^1^School of Medical Science and Griffith Health Institute, Griffith University, Gold Coast Campus, Southport, QLD 4222, Australia; ^2^School of Physiotherapy, Australian Catholic University, McAuley Campus, Banyo, QLD 4014, Australia; ^3^Rega Institute for Medical Research, KU Leuven, Minderbroedersstraat 10, 3000 Leuven, Belgium; ^4^School of Medicine, Deakin University, Waurn Ponds, VIC 3217, Australia

## Abstract

Solid tumour accounts for 90% of all cancers. The current treatment approach for most solid tumours is surgery, however it is limited to early stage tumours. Other treatment options such as chemotherapy and radiotherapy are non-selective, thus causing damage to both healthy and cancerous tissue. Past research has focused on understanding tumour cells themselves, and conventional wisdom has aimed at targeting these cells directly. Recent research has shifted towards understanding the tumour microenvironment and it's differences from that of healthy cells/tissues in the body and then to exploit these differences for treatmeat of the tumour. One such approach is utilizing anaerobic bacteria. Several strains of bacteria have been shown to selectively colonize in solid tumours, making them valuable tools for selective tumour targeting and destruction. Amongst them, the anaerobic *Clostridium* has shown great potential in penetration and colonization of the hypoxic and necrotic areas of the tumour microenvironment, causing significant oncolysis as well as enabling the delivery of therapeutics directly to the tumour *in situ*. Various strategies utilizing *Clostridium* are currently being investigated, and represent a novel area of emerging cancer therapy. This review provides an update review of tumour microenvironment as well as summary of the progresses and current status of Clostridial spore-based cancer therapies.

## 1. Introduction

Cancer is currently a major cause of morbidity and mortality internationally and poses a significant burden on both patients and their families and the healthcare system as a whole. Solid tumours, in particular, account for 90% of all cancers. A solid tumour is composed of a complex mix of tumour cells and nontumour cells, including supporting stromal and infiltrating blood cells, immune cells, and various molecules in proximity to these cells. This collection of cells and their metabolism is referred to as the tumour microenvironment. This unique tumour milieu not only allows for growth and metastasis, but additionally aids in the resistance of cancer cells to current chemotherapy and radiotherapy, thereby hindering their success. Consequently, there is an urgent demand for more suitable and effective treatment options for those suffering from solid tumours.

## 2. Tumour Microenvironment

Research in the past has typically focused on understanding the molecular and genetic aspects of cancer cells, which led to treatment options geared towards killing the cancer cells themselves. Recent advances in the field of oncology have lead to a greater understanding of tumour pathology. Currently, a greater research emphasis has been placed on comprehending the unique environment of solid tumours, referred to as the tumour microenvironment. Developments in this field have lead to the possibility of pursuing methods of treatment which serve to control this milieu in such a way that will enable us to manage the growth and metastasis of the tumour itself. This has generated the potential for new therapeutic targets and treatment options for cancer patients. *Clostridium*-spore-based bacterial therapy is one such a novel strategy.

### 2.1. Angiogenesis

 Growth and progression of tumour cells requires an increased supply of both oxygen and nutrients. For this the formation of a new vascular network is required to meet these demands [1]. Under regular physiological conditions, angiogenesis in cells results in a structurally well organized and highly efficient network of vasculature. However, this is not the case in tumours, where angiogenesis leads to a chaotic network of blood vessels. This is a distinctive component of the cancer microenvironment. This vasculature may be characterized by disorganization, dilation, branching, shunts, and varied diameters which results in inconsistent blood flow and further alters the microenvironment by creating hypoxic areas and regions high in acidity [1, 2]. Angiogenesis is a crucial component in the metastasis of cancer cells, and if it can be controlled, has the capability of halting the spread of cancer to other tissues. Additionally, the chaotic nature of the blood vessel and its network in the tumour microenvironment poses a problem for current cancer therapies in that it makes it difficult to administer drugs uniformly and in effective concentrations in all areas of the tumour. These are two problems which are trying to be overcome by instead treating the tumour environment and controlling angiogenesis as opposed to killing the cancer cells directly.

 The key mediator of angiogenesis is the glycoprotein vascular endothelial growth factor (VEGF), which stimulates blood vessel growth by acting on endothelial cells [3]. Additionally, VEGF can afford viability of immature blood vessels by preventing apoptosis, and evidence has shown it can also make vasculature more permeable causing leaky cell walls resulting in an increase of interstitial pressure [4–6]. Mediation by the VEGF proteins is a critical component in creating the chaotic vasculature of the tumour microenvironment, and as such is a primary research target for new therapeutic strategies.

### 2.2. Hypoxia and HIF-1

 Acute and chronic lack of blood supply resulting in the absence of oxygen and known as hypoxia is also a definitive characteristic of the tumour micro milieu and is caused by inefficient blood supply to cancerous cells. The hypoxic environment poses a challenge to traditional cancer treatment options in that this oxygen-depleted environment makes the solid tumour cells less susceptible to killing with chemotherapy and radiation, less genetically stable (thus more difficult to predict its response to treatment), and resistant to apoptosis [7]. Since these cells are devoid of oxygen, carbohydrate metabolism occurs via glycolysis resulting in the production of lactic acid and decreasing the pH of the tumour environment. Critical in the control of tumour hypoxia are the hypoxia-inducible factors (HIFs), a family of transcription factors which serve in the regulation of mammalian response to the absence of oxygen. One of the targets of HIF-1 is VEGF, which is switched on in a hypoxic environment. VEGF then stimulates angiogenesis creating poor vasculature within the tumours, causing further hypoxia creating a positive feedback loop where both factors are stimulated. It is important to note that VEGF is not the only target of HIF-1, and in fact, more than 60 direct target genes have been identified, many playing crucial roles in cell survival, metabolism, and metastasis of cancer cells [8]. The crucial role that HIF transcription factors have in creating the tumour microenvironment makes these proteins potentially important cancer treatment targets.

### 2.3. Apoptosis and Necrosis

 The proinflammatory cytokine tumour necrosis factor alpha (TNF-*α*) plays a unique role in the tumour environment where it permits cellular communication and is capable of promoting both growth and necrosis. TNF-*α* acts in conjunction with many other molecules within the cell to perform these tasks. For instance, tumour necrosis is carried out first by endothelial cell apoptosis caused by deactivation of the integrin a_v_b_3_ and disruption of the interface with the extracellular matrix (ECM), followed by T-cell activation to remove remaining tumour cells [9, 10]. In a contradictory role to this, in low chronic doses, TNF-*α* promotes tumour growth, invasion, and metastasis. One way it accomplishes this is by the remodelling of tissue by inducing matrix metalloproteins (MPPs) [11]. TNF-*α* also activates nuclear factor-*κ*b (NF-*κ*b), a transcription factor, which stimulates the proliferation of tumour cells and regulates antiapoptotic genes, and thus can protect the cancerous cells from the apoptotic cascade induced by TNF-*α* [12]. NF-*κ*b has also hindered some forms of cancer treatment involving cytokines, chemotherapeutics, and radiation which have all been shown to activate NF-*κ*b, helping to prevent apoptosis [13]. Both TNF-*α* and NF-*κ*b can both be targets for future cancer therapies; TNF-*α* can be triggered to induce apoptosis, and NF-*κ*b can be inhibited to prevent tumour growth and spread, as well as aid in making solid tumour cells more susceptible to apoptosis by treatment with other therapeutic cancer agents.

### 2.4. Tumour Structure and Stromal and Interstitial Pressure

 Tumour structure greatly contributes to the microenvironment of cancer. The membrane structure, in particular, is an essential part of tumour invasion and metastasis. The extracellular matrix (ECM) is a key component to the tumour structure, and it comprises molecules essential to cell signalling [14]. Structural components of the ECM include fibrous elements, link proteins (fibronectin for example), and space-filling molecules, whereas the signalling components comprise the cytokines, growth factors, ions, and peptides [14]. Fibronectins, one of the most abundant structural proteins of the ECM, have additional functions in tumour cells aside from their structural roles and aid in regulation of adhesion, migration, differentiation, and proliferation [15]. Fibronectins can also serve in signalling as solid-phase ligands, further adding to the complexity of the tumour micro milieu [16]. Another set of structural components of the ECM that duals as signalling molecules are collagens. In solid tumours, collagen has sometimes shown to be upregulated, providing a rigid matrix aiding in cell growth. The primary role of collagen, however, is in anchoring cells to the membrane and aiding in regulation [17]. As such, both fibronectin and collagen are key regulators of tumour invasion, metastasis, growth, proliferation, and signalling.

 Additional regulators of structural elements of the tumour cell are essential in metastasis. For example, the steps involved in the spread of cancer between tissues include the degradation of basement membrane and ECM, modulation of cell adhesion molecules, followed by migration to adjacent tissue [18]. During degradation of the basement membrane and ECM, matrix metalloproteinases (MMPs) play a vital role. MMPs are a family of enzymes capable of degrading all parts of the ECM, as well as other nonmatrix substrates which also contribute to the tumour microenvironment and progression [19–21]. MMPs also play a crucial role in angiogenesis, where they degrade the cellular matrix surrounding endothelial cells making them a target for antiangiogenesis [18, 22]. Research has shown them to be involved in cell growth (both inhibitory and stimulatory), proliferation, and apoptotic prevention as well [14].

 The high interstitial fluid pressure compared to that of normal cells is yet another key defining element of the tumour milieu. This is the result of the chaotic vasculature of solid tumours as previously described, due to high vessel permeability and cell density outside the vessels, low lymphatic drainage, and poor perfusion [23]. This causes problems for the administration of cancer therapeutics, which may not successfully be able to reach all tissues in sufficient quantities due to transcapillary transport [24].

### 2.5. Cancer-Related Inflammation

 Inflammation is present in the microenvironment of some solid tumours prior to metastasis, and in others, an oncogenic change induces an inflammatory response which aids in the development of tumours [25]. This inflammation assists in proliferation, survival, angiogenesis, and metastasis, while simultaneously facilitating in avoiding the adaptive immune system, and disrupting signals from hormones and chemotherapeutics [25]. Therefore, molecules which coordinate these inflammatory responses are important constituents of the tumour micro milieu. Cytokines and chemokines are two classes of these regulatory components.

 Chemokine is a family of cytokines which play a vital role in the directing leukocyte migration to sites of infection and inflammation in the body. In cancer, aberrant chemokine and cytokine receptor production facilitates tumour growth by mediating migration of leukocytes to tumour cells and stimulating the release of growth factors, such as tumour transforming growth factor (TGF), and promoting angiogenesis [26]. Cytokines are components of the immune system which stimulate the generation of antitumour specific responses. Additionally, however, cytokines may influence carcinogenesis and metastasis by modifying the tumour phenotype [27]. A primary example is interleukin-2 (IL2), a pluripotent cytokine which augments innate immune responses such as activation of natural killer and lymphokine-activated killer cells, and neoplastic cell killing by MHC-restricted T-cell responses [28].

## 3. The Impact of Tumour Micro Milieu on Current Cancer Therapy

 Thus far, the development of successful cancer therapies has been hampered by various aspects of the tumour micro milieu. Primarily, the obstacles of angiogenesis, hypoxia, and heterogeneous cell arrangement must somehow be overcome in order to develop viable treatment strategies for solid tumours. Along with increased efforts to understand the tumour microenvironment, alternative cancer treatment strategies have, however, emerged which seek to utilize this environment to an advantage.

### 3.1. Selective Tumour Colonization

 As previously discussed, the unique microenvironment of solid tumour cells consists of a complex, disarrayed set of blood vessels due to tumour angiogenesis, which leads to hypoxic areas, varying pH, and inconsistent blood flow. This poses a problem to current cancer treatment strategies in that therapeutics cannot be evenly distributed to all tumour tissue in effective concentrations, hindering some conventional chemotherapeutics. Additionally, many anticancer drugs target rapidly dividing cells and ignore the stromal cells and other cells that make up a tumour and can often result in tumour regrowth. Hypoxia in tumour cells may allow for inhibited cell cycle progression and proliferation, thus rendering these types of treatments ineffective [29]. In ionizing radiation, DNA damage occurs, which under aerobic conditions becomes lethal due to fixation by oxygen. In the anaerobic environment of solid tumours, however, the DNA can be restored to its original condition and may remain unharmed [30, 31]. The hypoxia of the tumour is undesirable for treatment in that it contributes to a higher degree of malignancy in the cancer cells, whilst aiding in cell development and angiogenesis [18]. Severe hypoxia in tumours, not surprisingly, has therefore been linked to poor prognosis [32]. Other treatment strategies have also been rendered less effective due to this hypoxic environment. The initially very promising area of utilizing adenoviral vectors to treat cancer was thwarted by this tumour hypoxia, where it induced halting of G1 cells responsible for viral replication, resulting in less effective treatment [33, 34]. The hypoxic milieu negatively influenced treatment via retroviral vectors due to the phosphorylation of eIF2*α* leading to inhibition of translation [35].

 Bacterial-based cancer therapy using *Clostridium* spores offers a selective advantage in overcoming the obstacles of hypoxia and necrosis. *Clostridium* species, being strictly anaerobic will only colonize in areas devoid of oxygen, and when systematically injected, spores germinate and multiply in the hypoxic/necrotic areas of solid tumours [36]. *Clostridium*, although anaerobic, possesses the ability to sporulate, allowing them to remain dormant in environments where oxygen is present. However, when growth conditions are suitable (i.e., in the hypoxic/necrotic milieu of solid tumours), the *Clostridium* spores germinate and begin to colonize these areas. This aspect of *Clostridium* growth is being exploited for use in a number of various novel cancer treatment strategies currently being developed which utilize *Clostridium* as a vector to deliver therapeutics directly to the solid tumour site (Table 1). Clostridial vectors can be safely administered as spores, and their efficacy in delivering and secreting therapeutic proteins has been demonstrated in a number of preclinical trials.

### 3.2. *Clostridium*-Directed Enzyme Prodrug Therapy

 One novel treatment strategy, known as suicide gene therapy or gene-directed enzyme prodrug therapy, utilizes an enzyme which cleaves or modifies a prodrug such that cleavage results in the active, oncolytic form of the toxin. Using *Clostridium* species as a vector, known as *Clostridium* directed enzyme prodrug therapy (CDEPT), the bacterium can be genetically modified to express these proteins. When spores are administered systematically, the *Clostridium* selectively grows and colonizes in the tumour cells where it expresses the prodrug cleaving enzyme. When the therapeutic is then administered, it is only cleaved into its active component in the localized tumour environment, resulting in tumour cell specificity as opposed to the nonspecific targeting exhibited by most other current treatment options, such as radiation. Although other vector systems exist (primarily viral vectors), bacterial vectors, *Clostridia* in particular, convey benefits such as lower toxicity, higher safety, and nonexistence no restraints on the gene size to be delivered. Additionally, bacteria can be rendered inactive rather quickly by the administration of antibiotics further increasing the safety of these approaches.

 Several enzyme/prodrug combinations are currently available. The cytosine deaminase (CD) and 5-fluorocytosine (5FC) system was one of the first systems to be cloned into *Clostridium*. CD converts the prodrug 5FC into 5-fluorouracil (5FU), a cytotoxic compound. Another common combination is the nitroreductase (NT R) enzyme, which converts CB1954 to a DNA cross-linking agent [37, 38]. Although these enzymes have been successfully cloned into a number of *Clostridium* strains, *C. sporogenes* has shown the highest potential thus far. Injection of recombinant CD expressing *C. sporogenes* NCIMB10696 spores into tumour-bearing mice was successful in tumour-specific expression of CD [39]. Moreover, when this was combined with its prodrug 5FC, significant tumour growth delay was achieved. Promising results were also obtained with the NTR enzyme/Pr-104 prodrug combination as well. NTR was successfully transformed into and expressed by *C. sporogenes*, and* in vivo* studies where spores were injected into tumour-bearing mice showed significant tumour reduction [40].

### 3.3. *Clostridium* to Enhance the Immune System and Tumour Cell Recognition

 Another novel treatment being developed in cancer therapy is genetic engineering of bacteria that express an enzyme possessing direct cytotoxic actions, as opposed to having prodrug cleaving actions. As previously discussed, TNF-*α*, a cytokine, can act as a tumour regressor in high doses where it functions as a vasculotoxic agent and as such is being researched as one of these cytotoxic enzymes. Over a decade ago, Theys et al. [41] genetically engineered *C. acetobutylicum* DSM792 to express and secrete murine TNF-*α*. Although TNF was secreted and biologically active, colonization levels of recombinant *C. acetobutylicum* were low, and sufficient amounts of TNF to combat the tumour cells were not secreted, and therefore no therapeutic benefits were observed [41]. More recently, an attempt was made at increasing TNF-*α* expression and secretion levels in *C. acetobutylicum *by adjusting transcription, translation, and secretion processes; however, no significant advances were made and TNF-*α* expression remained constant [42]. For this protein to be excreted in levels high enough to act as an antitumour agent, ways must be developed which would either allow increased TNF-*α* production at the tumour site or utilized the synergistic effect of interleukin-2 and TNF-*α* [42, 43].

 Interleukin-2 (IL-2) is a cytokine which enhances the immune system's natural anticancer functions [42]. Administration of cytokines such as IL-2 to the tumour environment may stimulate the immune system to discern and assail the solid tumour cells; however caution must be taken as high systemic levels of IL-2 cause toxicity. This was the basis for the genetic modification of *C*. *acetobutylicum* DSM792 by Barbe et al. [42] in 2005 to express increased levels of IL-2. By introducing rat IL-2 into *Clostridium*, solid tumours were specifically targeted and sufficient levels of IL2 were produced and excreted to decrease tumours in mice, while avoiding the effects of systemic toxicity. It may be possible to make treatment of this sort more effective by combining with other cytokines, enzymes, or chemotherapy. Additionally, by combining IL-2-recombinant *Clostridium* with a vascular targeting agent, it is hypothesized that colonization of the bacteria will increase while also increasing the release of tumour antigens from cells that have become necrotic, thus increasing the antitumour response by immune cells [42].

### 3.4. *Clostridium*-Directed Antibody Therapy (CDAT)

 Another area in which this is being utilized is *Clostridium*-directed antibody therapy (CDAT) where *Clostridium* is modified to produce high specificity antibodies against tumour antigens. Recently, CDAT was used to target HIF1*α* cells using the variable domain of the heavy-chain subclass of antibodies (VHH). A VHH against HIF1*α*, which when expressed in mammalian cells binds and inhibits HIF activity [44], was introduced into *C. novyi-NT *by heterologous gene transfer. The VHH, when isolated from the *Clostridia*, retained its binding capacity and specificity for the target, and overall the study demonstrated successful conjugation, expression, and functionality of these antibodies [45]. Further research into codon usage and promoters of the VHH antibody gene must be performed, however, to increase expression levels in *Clostridia* in order to successfully utilize this method in future cancer therapies. The potential of this would be in that by targeting HIF1*α*, hypoxia in the solid tumour could be controlled, and subsequently, factors contributing to metastasis and invasion could be eliminated, thus impeding the spread of the cancerous cells. This type of treatment would be useful as a combined modality treatment, where Clostridia spores are administered and express VHH upon germination. Once VHH minimizes tumour hypoxia by targeting HIF1*α*, a second treatment such as chemotherapy or radiation may be applied, which would be more effective as a result of the decreased hypoxia.

Our laboratory has recently created a hybrid toxin that could be expressed and delivered using the clostridial system. This toxin utilises the high affinity of receptor binding fragment of *Clostridium perfringens enterotoxin *(CPE). CPE naturally binds to CLDN-4 through the C-terminal 30 amino acid. Taking advantage of the fact that CLDN-4 is overexpressed on a range of cancer cells, we thus constructed a cDNA comprising C-CPE and a fragment of exotoxin A(ETA') (C-CPE-ETA'). The recombinant C-CPE-ETA' fusion protein was shown to retain the specificity of binding to CLDN-4 and initiate rapid penetration into cytosol in five different CLDN-4-positive cancer cells (MCF7, A431, SW480, PC3, and DU145) but not to CLDN-4-negative cells (HELA, HUVEC). C-CPE-ETA' was strongly cytotoxic towards CLDN-4-positive cancer cell, as opposed to cells lacking CLDN-4 expression. Moreover, we have also demonstrated that the recombinant fusion protein had significant anticancer ability in CLDN-4-positive cancer models *in vivo*. Subcutaneously implanted MCF7 and SW480 xenograft tumours were significantly decreased or abolished after three repeated injection of the hybrid toxin [46].

### 3.5. Combined Bacteriolytic Therapy (COBALT)

 COBALT is a proposed method of cancer treatment which has been relatively successful thus far. In this type of therapy, a bacterium is engineered to exhibit antitumour properties, such as proteolytic enzymes, which is then administered in conjunction with other known cancer therapies to work synergistically and improve oncolysis. A new strain of *Clostridium*, *C. novyi-NT*, which expresses proteolytic proteins was recently developed which showed significant antitumour activity in mice. To identify and manufacture this strain, 26 strains of bacteria were tested for tumour colonisation efficiency, and *C. novyi* was particularly promising. However, this contained a lethal toxin, which was subsequently genetically removed, and the newly engineered strain was designated *C. novyi-NT* [47]. Systemic administration of *C. novyi-NT *spores destroys adjacent cancer cells while simultaneously prompting inflammatory action by the recruitment of cytokines, attracting neutrophils, monocytes, and lymphocytes which attack cancer cells [38]. In an attempt to increase the antitumour action of the bacteria, spores of this strain were administered alongside of known anticancer drugs. Very promising results were obtained when bacterial *C. novyi-NT* spores were administered in conjunction with microtubule-interacting chemotherapeutic agents such as vinorelbine and docetaxel, and in 2006 a phase 1 clinical trial was commenced. Unfortunately, the first study was terminated due to design problems, but a second attempt at a phase one clinical trial to test safety has recently started and is still in the stages of recruiting participants [http://www.clinicaltrials.gov/ct2/results?term=c.novyi-NT].

### 3.6. Liposome-Mediated Preferential Release of Drugs at Solid Tumour Site

 Genome analysis of *C-novyi-NT *has demonstrated that some of its oncolytic capabilities are due to the presence of several lipid degrading enzymes, which are highly expressed when *C. novyi-NT *is colonised in tumours [31]. In 2006, Cheong et al. [48], exploited this property of *C. novyi-NT *to release liposomal drugs within the tumour. It was hypothesised that since this species lyses red blood cells, its ability to disrupt membranes might be able to increase the release of liposome-encapsulated drugs directly at the site of the tumour. A form of doxorubicin incorporated within a liposome called Doxil was utilised, and promising results were obtained. Systemic injection of *C. novyi-NT* spores in conjunction with Doxil completely eliminated tumours in two models. Further analysis of protein fractions of the culture medium, as well as mass spectroscopy analysis, revealed that lipase was indeed the protein product secreted by *C. novyi-NT* which increased the effectiveness of the liposome-encapsulated drug. The significance of this study is that further research can be performed using other liposome encapsulated drugs with specific targeting and release within the tumour microenvironment [31, 48].

 Although theoretically very promising, there are still some obstacles that must be overcome in order for *Clostridium*-directed therapies to be developed as a viable treatment option for cancer patients. Clostridium species which are easily transformable, such as *C. acetobutylicum*, do not typically have high colonisation efficiencies, whereas species which typically colonise well in tumour cells, such as *C. sporogenes*,* C. oncolyticum*, and *C. novyi-NT *are not easily transformed. However, recent advancements in genetic engineering technologies have allowed for improvements in this area. Using a new method of conjugative transfer from *E. coli*, Theys et al. were able to successfully transform plasmid vectors at higher frequencies into these strains of *Clostridium*, opening many new doors in *Clostridium*-directed cancer therapies [49]. Keeping this in mind, there are still many areas which need to be improved before *Clostridium* can be successfully implemented in current cancer therapies, but research efforts continue and advances are being made regularly in this field.

## 4. Conclusions and Future Perspectives

*Clostridium*-based cancer therapy is a promising approach for the treatment of solid tumours. Recently, understanding the solid tumour microenvironment and its influence on cancer therapy has profoundly changed our thinking about cancer therapy. We have realised that the unique solid tumour micro milieu has been one of the greatest hindrances thus far in successful cancer treatment. The utilisation of anaerobic *Clostridium* species allows for a targeted and curative treatment by destroying tumour microenvironment first, creating opportunity for combinational therapies. In addition, numerous strategies involving the administration of *Clostridium* spores to selectively deliver cancer therapeutics directly to the site of the solid tumour are currently being developed, and in many cases, promising oncolytic capabilities have been demonstrated. However, successful implementation of this mode of therapy in clinical trials relies on developmenting and manufacturing a *Clostridium* species to have both high colonisation efficiency and expression and excretion of sufficient high levels of the therapeutic proteins. Improving genetic engineering methods to genetically modify bacteria and modulation of gene expression to yield maximum protein secretion are areas which may enhance this field. *Clostridium*-based cancer therapies are one of the most novel and promising methods of cancer treatment currently being researched. The ability of these *Clostridium* based modalities to selectively target the microenvironment has provided a firm foundation for which to build towards efficient, safe and effective cancer treatments for the future and to improve the prognosis and treatment of so many individual patients suffering from solid tumours, relieving the burdens of patients, their families and the healthcare systems.

## Figures and Tables

**Table 1:** Summary of current methods being researched in *Clostridium*-based cancer therapies.

Method	Premise	Target/drugs	Clostridium species being used	Reference
*Clostridium* directed enzyme prodrug therapy (CDEPT)	*Clostridium* is genetically engineered to express an enzyme which cleaves a prodrug into its cytotoxic form.	CD/F-FU	*C. sporogenes* *C. beijerinckii * *C. acetobutylicum*	[39] [49] [41]
		NTR/CB1954	*C. beijerinckii * *C. sporogenes*	[37] [40]
		NTR/PR-104		

Administration of cytokines/cytotoxic agents	*Clostridium* is used to deliver agents (cytokines) to either act directly cytotoxic to cells or enhance immune system response to tumour cells.	murine TNF*α*	*C. acetobutylicum*	[41, 50, 51]
		IL-2	*C. acetobutylicum*	[42]

*Clostridium* directed antibody therapy (CDAT)	*Clostridium* is modified to produce highly specific antibodies against tumour antigens.	VHH against HIF*α*	*C. novyi-NT*	[45]

Combined bacteriolytic therapy (COBALT)	*Clostridium* which demonstrate direct antitumour effects are administered in conjunction with other known cancer therapies to increase oncolysis.	*Clostridium*/ mitomycin C and cytotaxin* Clostridium*/vinorelbine or docetaxel	*C. novyi-NT* *C. novyi-NT *	[47] [52]

Release of liposomal encapsulated drugs	Species of *Clostridium* which secrete lipid-degrading enzymes are used for the targeted release of liposome-encapsulated drugs at the tumour site.	*Clostridium*/Doxil	*C. novyi-NT*	[48]
